# How Did COVID-19 Impact Smoking Habits in the Saudi Community?

**DOI:** 10.7759/cureus.61243

**Published:** 2024-05-28

**Authors:** Faisal A Alhazani, Abdullah S Alsultan, Riyadh F Alshehri, Faisal T Alayed, Firas O Alhussini, Abdulaziz M Albalawi, Sulaiman M AlZamel, Mashael K Al-Ghanem, Waleed M Alhuzaim

**Affiliations:** 1 College of Medicine, Imam Mohammad Ibn Saud Islamic University, Riyadh, SAU; 2 Department of Medicine, Imam Mohammad Ibn Saud Islamic University, Riyadh, SAU

**Keywords:** e-smoking, vaping, e-cigarettes, electronic cigarettes, sars-cov-2, coronavirus disease (covid-19), cigarette smoking, smoking and covid-19, smoking tobacco

## Abstract

Background/aims

Most countries have gone through lockdowns to varying degrees during the COVID-19 pandemic to reduce the spread of the disease. The successive pandemic waves have impacted the health system, imposing restrictions set by the government. This changed people’s daily life routines and they felt more socially isolated, which in turn had an impact on their mental health. Some factors were linked to the severity and outcome of COVID-19 on patients. One of these factors was smoking. This study was carried out to investigate the prevalence and impact of lockdown on smoking habits, as well as the changes in attitudes, behavior, and the rate of consumption before and after the government restrictions in the general population of Saudi Arabia.

Materials and methods

The present cross-sectional study was conducted on a sample of 921 participants from the general population of Saudi Arabia. Data were collected via an online questionnaire. A structured self-response questionnaire was given to the participants after institutional research ethical approval was obtained for the study.

Results

A total of 921 participants from the smoker population of Saudi Arabia were included in the study. The majority of participants were male (72.9%), and more than half were aged between 18 and 34 years (53.7%). Single individuals had a higher prevalence of increased smoking and a lower rate of quitting compared to married individuals. Participants with higher education levels were more likely to continue smoking at the same rate. While 40.5% of participants reported no change in their smoking rate during the pandemic, 15.4% reported a decrease, 39.0% reported an increase, and 5.1% reported quitting smoking. Participants who reported feeling more stressed during the pandemic had a higher prevalence of increased smoking. The majority of participants believed that smoking increased the risk of COVID-19 infection.

Conclusion

The study highlights the need for targeted smoking cessation interventions and support services during the pandemic, considering demographic factors, living arrangements, and psychological impact. Efforts should be made to raise awareness about the negative health consequences of smoking during the pandemic and provide resources for stress management and alternative coping strategies. These findings have important implications for public health interventions and policies in Saudi Arabia.

## Introduction

The coronavirus disease 2019 (COVID-19) was first seen at the end of 2019 in Wuhan, China. Its long incubation period and the highly contagious nature of the virus, as well as the extensive global networking with high travel activities all over the world, have led to a rapid pandemic spread of SARS-CoV-2. During the COVID-19 pandemic, most countries have gone through lockdowns to varying degrees to reduce the spread of COVID-19 and to give more time to healthcare facilities to become better prepared for the management and prevention of the disease [1,2].

The successive pandemic waves have impacted the health system, imposing a significant burden on healthcare institutions and the health workforce in the country. Restrictions were set by the government and were not voluntary by the people themselves. Thus, this changed people’s daily life routines and they felt more socially isolated, which in turn had an impact on their mental health. Some factors were linked to the severity and outcome of COVID-19 on patients. One of these factors was smoking. Tobacco use around the world is a major threat to global health, with numerous studies indicating that it reduces the immune system and increases the chances of cardiovascular and respiratory illnesses [3,4]. Although the association between SARS-CoV-2 infection and smoking is not greatly understood, there is much evidence supporting a direct association between COVID-19 infection and smoking severity [5,6], with tobacco consumers almost having double the risk of COVID-19 progression and death [7].

According to a systematic review by Vardavas et al. (2020), there is a statistically significant correlation between smoking status and the major outcomes of death, ventilator use, or admission to the intensive care unit (ICU) among COVID-19 patients [8]. According to the review, smokers were 1.4 times more likely than non-smokers to experience severe COVID-19 symptoms, and they were roughly 2.4 times more likely to be hospitalized, require mechanical ventilation, or pass away. Additionally, another systematic review and meta-analysis by Alqahtani et al. (2020) revealed that serious COVID-19 problems affected 22% of current smokers and 46% of ex-smokers in the papers included and that current smoking was linked to a higher mortality risk among COVID-19 patients [9]. In addition, a recent meta-analysis of 19 studies, which included a total of 11,590 confirmed COVID-19 patients, illustrated that smoking was associated with a two-fold increase in the risk of disease progression [7], especially in connection to COVID-19-related respiratory symptoms [10].

Meanwhile, in a number of studies, stress has increased during the course of lockdown, and due to the pandemic, increased anxiety, economic concerns, and social isolation could have increased. In return, these stressors may have led to the increased initiation of tobacco use by non-users and/or increased product use among current users [11,12]. Previous studies have linked tobacco smoking to anxiety and depressive mood [13,14]. The COVID-19 pandemic has had a negative impact, which has been linked to increasing smoking rates [8]. Smoking is an essential regulator for smokers' mood problems because nicotine produces immediate anxiolytic effects [15].

According to researchers, the COVID-19 pandemic's quarantine and social isolation policies may exacerbate substance abuse as well as psychological problems like stress, anxiety, and irritability [16,17]. Smokers frequently claim that they smoke because it lowers their stress levels, and increased stress has been connected to increases in smoking [18].

Additionally, those who were trying to quit smoking or had already quit successfully at the time of the lockdown might be in danger of relapsing because high stress has been linked to both failures to quit smoking and encouraging smoking relapse in people who have previously quit [19]. On the other hand, social isolation could have resulted in a decline in social smokers, that is, those who smoke predominantly in social settings [20]. The prevalence of smoking among males and females aged 16 to 18 years in Saudi Arabia is estimated to be 55.6% and 31.4%, respectively [21].

This study was carried out to investigate the prevalence and the impact of lockdown on smoking habits, as well as the changes in attitudes, behavior, and the rate of consumption before and after the government restrictions in the general population of Saudi Arabia.

## Materials and methods

Study design

The present cross-sectional study was conducted between April and December 2022 on a sample of 921 participants from the general population of Saudi Arabia. The study included males and females aged 18 years and older who were smoking during the governmental restrictions of the COVID-19 pandemic. The sample size was calculated by using the Raosoft web tool (Raosoft, Inc., Seattle, WA) with a smoking prevalence rate of 14% in the Kingdom of Saudi Arabia (KSA) [22]. Data were collected via an online questionnaire. Validation of the questionnaire was done via a pilot study on 15 individuals to verify the validity of the questions and the correctness of the language. This helped to obtain more accurate results for the questionnaire. The consent of all participants was obtained, all personal information was kept confidential, and the participants were assured that all collected data would be used only for scientific research purposes and remained highly secure.

A structured self-response questionnaire was given to the participants to assess many variables. The questionnaire evaluated a range of aspects, beginning with information regarding sociodemographic factors, such as age, gender, marital status, educational level, occupational status, and residential areas. According to their age, the participants were classified into three groups: 18-34 years, 35-54 years, and 55 years or above. According to their marital status, participants were categorized as single or married. Residential areas were grouped as northern, southern, eastern, western, and middle regions. The other section of the questionnaire was about smokers. The types of smoking were classified into cigarettes, e-cigarettes, and shisha. Participants were asked, "What are the symptoms that accompany smoking?" to assess the consequences of smoking, such as headache, dizziness, cough, insomnia, anxiety, or nervousness. Moreover, participants were asked, "How long have you been smoking?" and classified into one to three years, five to 10 years, and more than 10 years. Participants were transferred to another section if they had ever quit smoking and were asked, "When did you quit smoking?" to know if there was any relation to the pandemic. Also, participants were asked, "Have you been infected with SARS-2 coronavirus?" and "When did you get infected with SARS-2 coronavirus?" to assess if there was any change in smoking habits due to the disease. In relation to COVID-19, participants were asked "Were you smoking before the pandemic?" "Average smoking rate during the pandemic," and "Average smoking rate after the pandemic" to assess if the rate of consumption differed before and after the pandemic lockdown and the impact of the lockdown on smoking behavior.

Statistical analysis

After collecting data, Microsoft Excel (Microsoft Corporation, Redmond, WA) was used for data entry, cleaning, and coding, while SPSS version 26 (IBM Corp., Armonk, NY) was used for data analysis. Frequency and percentage were used for the description of categorical variables and presented as tables and figures. Chi test, t-test, and ANOVA tests were used to compare participants with different demographic characteristics and rates of smoking during and after the COVID-19 pandemic. All statements were considered significant when the p-value was lower than 0.05.

## Results

The study included a total of 921 participants from the general smokers of Saudi Arabia. The majority of participants were male (72.9%), and more than half of the participants were aged between 18 and 34 years (53.7%). Regarding marital status, 48.2% of the participants were single, while 51.8% were married. In terms of occupation, most of the participants were classified as governmental or private sector employees (27.1% and 26.9%, respectively), and 24.4% were students. The participants were also categorized based on their residential areas, with the majority residing in the central region (46.8%), followed by the western region (20.8%) and the eastern region (14.2%). In terms of educational level, most of the participants reported having a college education level (54.9%). The majority of participants (85.2%) reported living with their families, while 14.8% lived alone. In terms of housing type, most participants lived in villas (70.0%), followed by apartments (30.0%) (Table 1).

**Table 1:** Demographic factors of the participants (N = 921). The data have been presented as count and N (%).

		Count	N (%)
Gender	Male	671	72.9%
	Female	250	27.1%
Age	18-34	495	53.7%
	35-54	301	32.7%
	55 or older	125	13.6%
Marital status	Single	444	48.2%
	Married	477	51.8%
Occupation	Unemployed	96	10.4%
	Students	225	24.4%
	Government employee	250	27.1%
	Private sector employee	248	26.9%
	Retired	102	11.1%
Residency	Northern region	73	7.9%
	Southern region	94	10.2%
	Central region	431	46.8%
	Eastern region	131	14.2%
	Western region	192	20.8%
Educational level	Less than primary school	25	2.7%
	Primary/intermediate school	37	4.0%
	High school	232	25.2%
	College	506	54.9%
	Higher education	121	13.1%
Living with	Alone	136	14.8%
	Family	785	85.2%
Type of home	Apartment	276	30.0%
	Villa	645	70.0%

**Figure 1:**
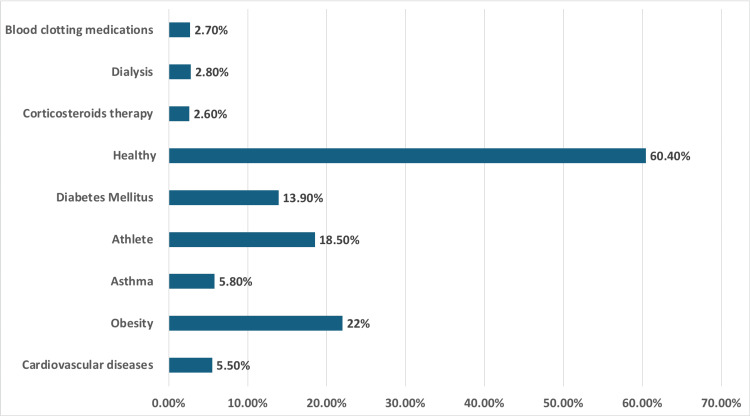
Health/medical status of the patients. The data have been presented as N (%).

Among the participants, 60.4% were healthy. The most prevalent health/medical status reported was obesity (22.0%), followed by diabetes mellitus (13.9%), athletes (18.5%), asthma (5.8%), cardiovascular diseases (5.5%), dialysis (2.8%), corticosteroid treatment (2.6%), and blood clotting medications (2.7%) (Figure 1).

Regarding smoking characteristics, the participants reported smoking different types of tobacco products: cigarettes (47.1%), shisha (27.3%), and electronic cigarettes (25.6%). In terms of the duration of smoking, 8.3% of participants reported having stopped smoking, while 19.7% reported smoking for one to three years, 40.1% for five to 10 years, and 32.0% for more than 10 years. Among those who quit smoking, the majority stopped in 2020 (38.2%), followed by 2022 (38.2%) and 2021 (23.7%). In terms of COVID-19 diagnosis, 50.7% of participants reported being diagnosed with COVID-19. Among those diagnosed with COVID-19, 82.4% reported that COVID-19 affected their mental health. The majority of participants who caught COVID-19 did so in 2020 (52.7%), followed by 2021 (34.5%) and 2022 (12.8%). The severity of COVID-19 symptoms varied, with 36.0% reporting flu-like symptoms, 48.2% reporting severe symptoms treated at home, 11.8% reporting severe symptoms treated at the hospital, and 4.1% reporting severe symptoms treated at the ICU (Table 2).

**Table 2:** Smoking characteristics and COVID-19-related disorders. The data have been presented as count and N (%).

		Count	N (%)
Type of smoking?	Cigarette	434	47.1%
	Shisha	251	27.3%
	Electronic cigarette	236	25.6%
Duration of smoking?	Stopped smoking	76	8.3%
	1-3 years	181	19.7%
	5-10	369	40.1%
	>10 years	295	32.0%
Year of quitting smoking?	2020	29	38.2%
	2021	18	23.7%
	2022	29	38.2%
Have you been infected with COVID-19?	No	454	49.3%
	Yes	467	50.7%
COVID-19 affected mental health?	No	162	17.6%
	Yes	759	82.4%
Year of catching COVID-19?	2020	246	52.7%
	2021	161	34.5%
	2022	60	12.8%
Severity?	Flu-like symptoms	168	36.0%
	Severe symptoms treated at home	225	48.2%
	Severe symptoms treated at the hospital	55	11.8%
	Severe symptoms treated at ICU	19	4.1%

**Figure 2:**
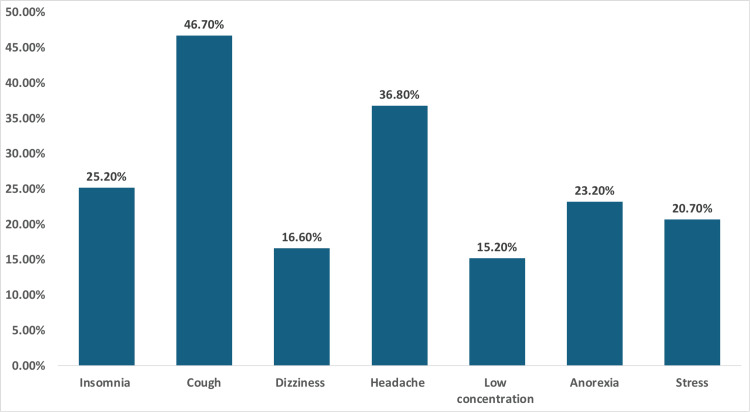
Medical conditions associated with smoking. The data have been presented as N (%).

Participants reported various health issues associated with smoking, including stress (20.7%), anorexia (23.2%), low concentration (15.2%), headache (36.8%), dizziness (16.6%), cough (46.7%), and insomnia (25.2%) (Figure 2).

Before the COVID-19 pandemic, the majority of participants (89.1%) reported being smokers. During the pandemic, 40.5% reported no change in their smoking rate, 15.4% reported a decrease, 39.0% reported an increase, and 5.1% reported quitting smoking. After the pandemic, 53.5% reported no change in their smoking rate, 14.7% reported a decrease, 22.7% reported an increase, and 9.1% reported quitting smoking. Regarding the psychological impact of the pandemic on the desire to quit smoking, 50.3% of participants reported no change, 16.6% reported a decrease, 26.7% reported an increase, and 6.4% reported quitting smoking. Participants who were healthy accounted for 49.7% of the sample. Among those with a medical issue, 25.0% reported no change in their smoking behavior, 5.3% reported a decrease, 14.9% reported an increase, and 5.1% reported quitting smoking. In addition, 27.8% of the participants thought that smoking could increase the possibility of infection with COVID-19, while only 4.6% thought that smoking had protective effects against infections. Moreover, 17.7% and 5.4% of the participants thought that smoking at home or in public places was safe. In cases of infections, 50.7% of the participants thought that smoking would worsen the cases (Table 3).

**Table 3:** The change in smoking rates during and after the COVID-19 pandemic and the investigation of the different factors affecting this change. The data have been presented as count and N (%).

		Count	N (%)
Were you smoking before COVID-19?	No	100	10.9%
	Yes	821	89.1%
Smoking rate during the COVID-19 pandemic?	Unchanged	373	40.5%
	Decreased	142	15.4%
	Increased	359	39.0%
	Stopped smoking	47	5.1%
Smoking rate after the COVID-19 pandemic?	Unchanged	493	53.5%
	Decreased	135	14.7%
	Increased	209	22.7%
	Stopped smoking	84	9.1%
The psychological impact of the pandemic on the desire to quit smoking?	Unchanged	463	50.3%
	Decreased	153	16.6%
	Increased	246	26.7%
	Stopped smoking	59	6.4%
The impact of chronic diseases on smoking behavior during the pandemic?	Healthy	458	49.7%
	Unchanged	230	25.0%
	Decreased	49	5.3%
	Increased	137	14.9%
	Stopped smoking	47	5.1%
The rate of fear of respiratory diseases due to smoking coinciding with the pandemic?	Unchanged	455	49.4%
	Decreased	47	5.1%
	Increased	367	39.8%
	Stopped smoking	52	5.6%
Do you think that smoking increases the possibility of infection with coronavirus?	No	180	19.5%
	Yes	256	27.8%
	I do not know	485	52.7%
Do you think that smoking protects against infection with coronavirus?	No	654	71.0%
	Yes	42	4.6%
	I do not know	225	24.4%
What do you think about smoking at home during the pandemic?	No safe	549	59.6%
	Safe	163	17.7%
	I do not know	209	22.7%
What do you think about smoking in public places during the pandemic?	No safe	728	79.0%
	Safe	50	5.4%
	I do not know	143	15.5%
What do you think will be the outcome for a smoker when infected with coronavirus?	As non-smokers	145	15.7%
	Worsen	467	50.7%
	Fast recovery	44	4.8%
	I do not know	265	28.8%

During the pandemic, smoking rates were influenced by various demographic factors. In terms of gender, 42.9% of males and 34.0% of females maintained the same smoking rate as before, while 13.0% of males and 22.0% of females decreased smoking, and this difference is significant (P = 0.000). Age also played a significant role, with older participants having a higher tendency to reduce, quit, or at least smoke at the same rate, while younger participants had a higher risk of increasing their smoking rate (P = 0.000). Marital status also showed significant variations, as 32.7% of single individuals had the same smoking rate, 13.7% decreased smoking, 48.2% increased smoking, and 5.4% stopped smoking compared with married individuals, where 47.8% had the same smoking rate, 17.0% decreased smoking, 30.4% increased smoking, and 4.8% quit smoking. In addition, unemployed participants and students reported an increased rate of smoking compared to employees who showed either constant rates or lower rates of smoking during COVID-19. In addition, those who had a college education showed a higher tendency to increase their smoking rate during the pandemic compared to other educational levels (46.0%, P = 0.000). Moreover, living alone or in an apartment increases the risk of increasing the rate of smoking during the COVID-19 pandemic. Furthermore, not having any health/medical issue is associated with a higher rate of increasing the rate of smoking significantly (42.4% vs. 33.7%, P = 0.032) while being diagnosed with COVID-19 increases the risk of increasing the rate of smoking (42.4% vs. 35.5%, P = 0.000) (Table 4).

**Table 4:** The relation between demographic factors and smoking rates during the COVID-19 pandemic. The data have been presented as N (%) and p-value. * P-value < 0.05 is considered significant.

		Smoking rate during the COVID-19 pandemic								
		Unchanged		Decreased		Increased		Stopped smoking		P-value
		N	%	N	%	N	%	N	%	
Gender	Male	288	42.9%	87	13.0%	271	40.4%	25	3.7%	0.000*
	Female	85	34.0%	55	22.0%	88	35.2%	22	8.8%	
Age	18-34	170	34.3%	84	17.0%	218	44.0%	23	4.6%	0.000*
	35-54	137	45.5%	46	15.3%	104	34.6%	14	4.7%	
	55 or older	66	52.8%	12	9.6%	37	29.6%	10	8.0%	
Marital status	Single	145	32.7%	61	13.7%	214	48.2%	24	5.4%	0.000*
	Married	228	47.8%	81	17.0%	145	30.4%	23	4.8%	
Occupation	Unemployed	31	32.3%	13	13.5%	46	47.9%	6	6.3%	0.000*
	Students	77	34.2%	30	13.3%	112	49.8%	6	2.7%	
	Government employee	121	48.4%	45	18.0%	74	29.6%	10	4.0%	
	Private sector employee	92	37.1%	46	18.5%	93	37.5%	17	6.9%	
	Retired	52	51.0%	8	7.8%	34	33.3%	8	7.8%	
Residency	Northern region	31	42.5%	14	19.2%	24	32.9%	4	5.5%	0.036*
	Southern region	29	30.9%	12	12.8%	44	46.8%	9	9.6%	
	Central region	173	40.1%	53	12.3%	186	43.2%	19	4.4%	
	Eastern region	58	44.3%	26	19.8%	41	31.3%	6	4.6%	
	Western region	82	42.7%	37	19.3%	64	33.3%	9	4.7%	
Educational level	Less than primary school	14	56.0%	4	16.0%	6	24.0%	1	4.0%	0.000*
	Primary/intermediate school	23	62.2%	8	21.6%	6	16.2%	0	0.0%	
	High school	93	40.1%	40	17.2%	88	37.9%	11	4.7%	
	College	171	33.8%	76	15.0%	233	46.0%	26	5.1%	
	Higher education	72	59.5%	14	11.6%	26	21.5%	9	7.4%	
Living with	Alone	41	30.1%	20	14.7%	67	49.3%	8	5.9%	0.032*
	Family	332	42.3%	122	15.5%	292	37.2%	39	5.0%	
Type of home	Apartment	101	36.6%	51	18.5%	115	41.7%	9	3.3%	0.065
	Villa	272	42.2%	91	14.1%	244	37.8%	38	5.9%	
Presence of health/medical issue	No	206	37.1%	88	15.8%	236	42.4%	26	4.7%	0.032*
	Yes	167	45.7%	54	14.8%	123	33.7%	21	5.8%	
Have you been infected with COVID-19?	No	210	46.3%	53	11.7%	161	35.5%	30	6.6%	0.000*
	Yes	163	34.9%	89	19.1%	198	42.4%	17	3.6%	

The smoking rate after the COVID-19 pandemic varied based on different demographic factors. Gender played a significant role, with 58.1% of males and 41.2% of females continuing to smoke at the same rate. In terms of age, 47.9% of individuals aged 18-34 years, 60.5% of individuals aged 35-54 years, and 59.2% of individuals aged 55 years or older maintained their previous smoking habits. Marital status also influenced smoking rates, as 48.2% of single individuals and 58.5% of married individuals continued smoking at the same rate. Occupation played a role as well, with 42.7% of non-working individuals, 48.0% of students, 60.8% of government employees, 52.8% of private sector employees, and 59.8% of retired individuals maintaining their previous smoking rate. The region of residence did not show a significant association with smoking rates after the pandemic. Educational level had an impact, as individuals with higher education levels were more likely to continue smoking at the same rate. Living arrangements also played a role, with 44.9% of individuals living alone and 55.0% of those living with family members maintaining their previous smoking habits. The type of home had a significant association, as 50.0% of individuals living in apartments and 55.0% of those living in villas continued smoking at the same rate. Health/medical issues and a previous COVID-19 diagnosis did not show a strong relationship with smoking rates after the pandemic (Table 5).

**Table 5:** The relation between demographic factors and smoking rate after the COVID-19 pandemic. The data have been presented as N (%) and p-values. * P-value < 0.05 is considered significant.

		Smoking rate after the COVID-19 pandemic								
		Unchanged		Decreased		Increased		Stopped smoking		P-value
		N	%	N	%	N	%	N	%	
Gender	Male	390	58.1%	86	12.8%	144	21.5%	51	7.6%	0.000*
	Female	103	41.2%	49	19.6%	65	26.0%	33	13.2%	
Age	18-34	237	47.9%	73	14.7%	138	27.9%	47	9.5%	0.001*
	35-54	182	60.5%	44	14.6%	53	17.6%	22	7.3%	
	55 or older	74	59.2%	18	14.4%	18	14.4%	15	12.0%	
Marital status	Single	214	48.2%	57	12.8%	131	29.5%	42	9.5%	0.000*
	Married	279	58.5%	78	16.4%	78	16.4%	42	8.8%	
Occupation	Unemployed	41	42.7%	17	17.7%	30	31.3%	8	8.3%	0.000*
	Students	108	48.0%	30	13.3%	71	31.6%	16	7.1%	
	Government employee	152	60.8%	40	16.0%	38	15.2%	20	8.0%	
	Private sector employee	131	52.8%	33	13.3%	58	23.4%	26	10.5%	
	Retired	61	59.8%	15	14.7%	12	11.8%	14	13.7%	
Residency	Northern region	37	50.7%	14	19.2%	17	23.3%	5	6.8%	0.232
	Southern region	48	51.1%	12	12.8%	22	23.4%	12	12.8%	
	Central region	245	56.8%	49	11.4%	93	21.6%	44	10.2%	
	Eastern region	67	51.1%	27	20.6%	30	22.9%	7	5.3%	
	Western region	96	50.0%	33	17.2%	47	24.5%	16	8.3%	
Educational level	Less than primary school	10	40.0%	3	12.0%	10	40.0%	2	8.0%	0.002*
	Primary/Intermediate school	28	75.7%	4	10.8%	5	13.5%	0	0.0%	
	High school	115	49.6%	44	19.0%	53	22.8%	20	8.6%	
	College	259	51.2%	73	14.4%	126	24.9%	48	9.5%	
	Higher education	81	66.9%	11	9.1%	15	12.4%	14	11.6%	
Living with	Alone	61	44.9%	31	22.8%	36	26.5%	8	5.9%	0.006*
	Family	432	55.0%	104	13.2%	173	22.0%	76	9.7%	
Type of home	Apartment	138	50.0%	63	22.8%	57	20.7%	18	6.5%	0.000*
	Villa	355	55.0%	72	11.2%	152	23.6%	66	10.2%	
Presence of health/medical issue	No	286	51.4%	79	14.2%	139	25.0%	52	9.4%	0.013*
	Yes	207	56.7%	56	15.3%	70	19.2%	32	8.8%	
Have you been infected with COVID-19?	No	255	56.2%	50	11.0%	103	22.7%	46	10.1%	0.016*
	Yes	238	51.0%	85	18.2%	106	22.7%	38	8.1%	

## Discussion

The primary objective of this study was to examine the prevalence and consequences of the implementation of lockdown measures on smoking patterns, as well as shifts in attitudes, behaviors, and consumption rates among the general population of Saudi Arabia. The results of this study offer significant contributions to our understanding of smoking patterns during the COVID-19 epidemic, elucidating the impact of several demographic variables on smoking prevalence.

The research encompassed a sample size of 921 individuals who were representative of the general smoking population in Saudi Arabia. The male gender constituted the majority of participants (72.9%), aligning with prior studies that have demonstrated an elevated prevalence of smoking among males in Saudi Arabia [22,23]. The observed discrepancy in smoking prevalence across genders can be attributed to cultural standards, societal influences, and specialized marketing tactics employed by tobacco corporations [24]. The significant representation of male participants in the study indicates the need for the development of smoking cessation therapies that are specifically designed to address the unique needs and characteristics of males in the context of Saudi Arabia.

The analysis of participants' age distribution indicated that a majority of the participants fell between the age range of 18 and 34 years, accounting for 53.7% of the total sample. The age demographic under consideration plays a crucial role in the implementation of smoking prevention and cessation strategies since the early adulthood phase is more susceptible to the initiation and establishment of smoking behaviors [25]. The observation that younger individuals exhibited a greater propensity for elevating their smoking habits within the epidemic in comparison to their older counterparts is a matter of concern. The aforementioned discovery emphasizes the necessity of implementing focused interventions and educational initiatives aimed at increasing knowledge regarding the adverse consequences of smoking and the potential influence of the pandemic on smoking habits among younger demographics.

The study revealed a significant correlation between marital status and smoking prevalence throughout the pandemic. The findings of the study indicated that there was a greater prevalence of heightened smoking habits among single individuals, whereas married individuals had a reduced propensity for smoking cessation. Previous research has revealed similar findings, indicating that marriage may have a preventive effect against smoking [26,27].

The occupation of individuals was found to be a contributing factor to variations in smoking rates during the pandemic. Students were found to have a higher tendency to increase their rate of smoking during the pandemic. The observed results could potentially be linked to factors such as levels of stress, the prevailing culture within the workplace, and the availability of tools for smoking cessation. Conversely, students who had disruptions in their scholastic routines as a result of the epidemic may have been afforded increased opportunities for introspection over their smoking habits and subsequent modifications [28].

There was an observed correlation between educational attainment and smoking prevalence during the pandemic. Individuals with higher levels of education exhibited a greater propensity to maintain their smoking habits at a consistent frequency. This discovery is in disagreement with prior studies that have demonstrated a negative correlation between greater education levels and increased rates of smoking [29].

The investigation also analyzed alterations in smoking habits prior to, during, and subsequent to the pandemic. It is of significance to note that a considerable proportion of participants, specifically 40.5%, said that their smoking rate remained unchanged during the course of the pandemic. Additionally, 15.4% of individuals reported a drop in their smoking habits, while 39.0% reported an increase. Furthermore, a smaller percentage of participants, specifically 5.1%, reported that they had successfully quit smoking. The aforementioned findings suggest that the COVID-19 pandemic has elicited a varied influence on smoking patterns within the population of Saudi Arabia. While a subset of participants showed success in reducing or ceasing their smoking habits, a notable proportion observed an elevation in their smoking rates. These results are similar to those reported previously by different studies [30-32].

The pandemic's psychological ramifications have also exerted an influence on smoking habits. Individuals who indicated experiencing higher levels of stress during the pandemic exhibited a greater incidence of heightened smoking behavior. This discovery aligns with prior studies that propose individuals may resort to smoking as a means of coping during periods characterized by stress and ambiguity [19,33]. The examination of the psychological dimensions of smoking and the provision of alternative coping mechanisms are of paramount importance in addressing the needs of individuals who are confronted with elevated levels of stress.

The investigation additionally evaluated the individuals' perspectives on smoking during the ongoing pandemic. A significant proportion of the participants (79.2%) held the belief that smoking is associated with an elevated likelihood of infection with COVID-19, hence demonstrating a considerable level of consciousness regarding the adverse health implications of smoking amidst the ongoing epidemic. Nevertheless, even at this level of consciousness, a notable section of the subjects nevertheless indicated an escalation in their smoking habits. This implies that further endeavors are required to narrow the disparity between knowledge acquisition and behavioral modification, such as facilitating the availability of smoking cessation tools and advocating for the establishment of smoke-free settings.

It is imperative to acknowledge that this study possesses many limitations. Initially, the research was dependent on data that were self-reported, potentially introducing recollection bias and social desirability bias. Furthermore, the research conducted in this study specifically targeted the overall population of Saudi Arabia. Consequently, it is important to note that the results obtained may not be applicable to particular subgroups, such as persons who already have mental health disorders or those who live in specific regions characterized by distinct smoking habits. Subsequent investigations ought to acknowledge these constraints and offer a more exhaustive comprehension of smoking patterns amidst the COVID-19 epidemic.

## Conclusions

In conclusion, this research offers significant findings about the frequency and consequences of the pandemic on smoking habits within the context of Saudi Arabia. The results underscore the necessity of implementing smoking cessation therapies, educational initiatives, and policies that take into account the impact of demographic variables, living situations, and psychological aspects on smoking prevalence. Through the consideration of these many elements and the provision of appropriate resources and assistance, public health initiatives have the potential to effectively decrease smoking prevalence and alleviate the adverse health outcomes linked to smoking in times of crisis.
